# The complete mitochondrial genome of the *Alticorpus geoffreyi*

**DOI:** 10.1080/23802359.2016.1176876

**Published:** 2016-06-20

**Authors:** Da-Shi Qi, Jin-hao Tao, Xiao-Jing Huang, Jie Jiang, Mei Wang, Lian-Qin Zhang, Xin-Yu Mei

**Affiliations:** a Department of Genetics, Xuzhou Medical College, Xuzhou, People’s Republic of China;; b Interdisciplinary Center on Biology and Chemistry, Shanghai Institute of Organic Chemistry, Chinese Academy of Sciences, Shanghai, People’s Republic of China;; c Pediatric Emergency and Critical Care Center, Children' Hospital of Fudan University, Shanghai, People’s Republic of China

**Keywords:** *Alticorpus geoffreyi*, mitogenome, phylogenic relationship

## Abstract

The present study reported the complete mitochondrial genome of *Alticorpus geoffreyi* for the first time. The mitochondrial genome of *A. geoffreyi* possesses 16,578 bp in length, involving 22 transfer RNA genes, 2 ribosomal RNA, 13 protein-coding genes and one control region. In addition, its GC content is 45.82% that is similar to that of *Astatotilapia calliptera* (the GC content of 45.90%). Based on the complete mitochondrial genomes of 14 closely related species, the phylogenetic tree was further made to show their phylogenic relationship. The results will serve as a useful dataset for studying the evolution of Cichlidae mitochondrial genome.

The *Alticorpus geoffreyi* is a member of Cichlidae native to the Southeast Arm of Lake Malawi. Our present study reported the complete mitochondrial genome of *A. geoffreyi* for the first time, which would facilitate our understanding of the genetics, systematics and phylogenetic relationships of the many species of Cichlidae family.

The complete mitochondrial genome of *A. geoffreyi* (GenBank accession NC_028033) was assembled based on its raw sequences of whole genome (GenBank accession ERP002088) (Tan et al. 2015). The whole genome of this species was sequenced by the Wellcome Trust Sanger Institute (SC) in the project PRJEB1254 (NCBI accession number), and the accession number of the sample on NCBI is SAMEA1904331. Additionally, its complete mitochondrial genome sequence was also annotated by using DOGMA (Alverson et al. 2010).

The entire mitochondrial genome of *A. geoffreyi* possesses 16,578 bp, involving 22 transfer RNA (tRNA) genes, 2 ribosomal RNA (rRNA) genes, 13 protein-coding genes (PCGs) and one control region. 28 genes including 12 PCGs, 14 tRNA and 2 rRNA are H-strand, whereas 9 genes including 1 PCG and 8 tRNA are L-strand. 11 PCGs had ATG start codon except for cox1 with GTG and nd6 with TTA. Three complete stop codons (TAA, TAG and CAT) and one incomplete stop codon (T– –) were found in the PCGs in *A. geoffreyi*. The 22 tRNA genes begin from 67 bp (tRNA^Ser^, tRNA^Cys^) to 74bp (tRNA^Leu^), while 16S rRNA possesses 1675 bp and 12S rRNA possesses 941 bp in length. In addition, its GC content is 45.82% (27.58% A, 26.60% T, 30.15% C and 15.67% G) that is similar to that of *Astatotilapia calliptera* (the GC content of 45.90%). Based on the complete mitochondrial genomes of the *A. geoffreyi*, *A. calliptera* (GenBank accession no. NC_018560), and other 12 closely related species, we also used MEGA6.06 to construct the phylogenetic tree by Maximum likelihood method (Figure 1; Stamatakis et al. 2002; Tamura et al. 2013; Qi et al. 2015). Those results would facilitate our understanding of the evolution of Cichlidae mitochondrial genome.

**Figure 1.**
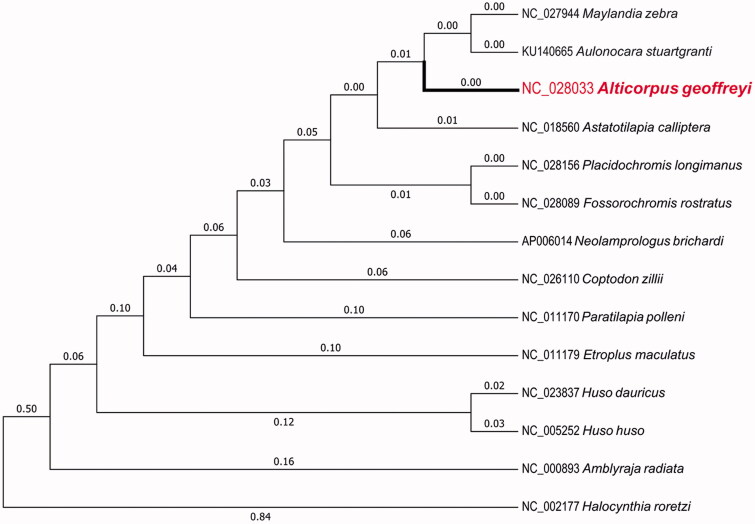
Maximum likelihood tree of complete mitochondrial genome of *A. geoffreyi* and 13 other closely species, which have complete mitochondrial genome sequences in NCBI. The numbers in front of the species are GenBank accession numbers.
